# Linking Emergency Medical Services and Emergency Department Data to Improve Overdose Surveillance in North Carolina

**DOI:** 10.1177/00333549211012400

**Published:** 2021-11-02

**Authors:** Jonathan Fix, Amy I. Ising, Scott K. Proescholdbell, Dennis M. Falls, Catherine S. Wolff, Antonio R. Fernandez, Anna E. Waller

**Affiliations:** 1 2331 484049 Department of Epidemiology, University of North Carolina, Chapel Hill, NC, USA; 2 Department of Emergency Medicine, University of North Carolina, Chapel Hill, NC, USA; 3 114146 North Carolina Division of Public Health, Raleigh, NC, USA

**Keywords:** surveillance, data linkage, emergency medicine

## Abstract

**Introduction:**

Linking emergency medical services (EMS) data to emergency department (ED) data enables assessing the continuum of care and evaluating patient outcomes. We developed novel methods to enhance linkage performance and analysis of EMS and ED data for opioid overdose surveillance in North Carolina.

**Methods:**

We identified data on all EMS encounters in North Carolina during January 1–November 30, 2017, with documented naloxone administration and transportation to the ED. We linked these data with ED visit data in the North Carolina Disease Event Tracking and Epidemiologic Collection Tool. We manually reviewed a subset of data from 12 counties to create a gold standard that informed developing iterative linkage methods using demographic, time, and destination variables. We calculated the proportion of suspected opioid overdose EMS cases that received *International Classification of Diseases, Tenth Revision, Clinical Modification* diagnosis codes for opioid overdose in the ED.

**Results:**

We identified 12 088 EMS encounters of patients treated with naloxone and transported to the ED. The 12-county subset included 1781 linkage-eligible EMS encounters, with historical linkage of 65.4% (1165 of 1781) and 1.6% false linkages. Through iterative linkage methods, performance improved to 91.0% (1620 of 1781) with 0.1% false linkages. Among statewide EMS encounters with naloxone administration, the linkage improved from 47.1% to 91.1%. We found diagnosis codes for opioid overdose in the ED among 27.2% of statewide linked records.

**Practice Implications:**

Through an iterative linkage approach, EMS–ED data linkage performance improved greatly while reducing the number of false linkages. Improved EMS–ED data linkage quality can enhance surveillance activities, inform emergency response practices, and improve quality of care through evaluating initial patient presentations, field interventions, and ultimate diagnoses.

From 1999 to 2018, more than 446 000 deaths in the United States involved opioids.^1^ During that period, more than 14 500 unintentional opioid-involved overdose deaths occurred in North Carolina. Although the 6780 opioid overdose visits to an emergency department (ED) in North Carolina in 2018 represent a 7% reduction in visits from 2017, they reflect a nearly 130% increase compared with 2010.^2^ Naloxone has been an important tool in fighting the opioid overdose epidemic, and North Carolina has substantially increased access to and use of naloxone for opioid overdose reversal.^3^

Public health surveillance plays a critical role in revealing trends in opioid overdoses over time and identifying key populations at greater risk of opioid-related morbidity and mortality.^1,4^ Although emergency medical services (EMS) reports of naloxone administration have been used to estimate the number of suspected overdose cases, this surveillance method has limitations in correctly identifying cases.^5^ For patients treated with naloxone by EMS and transported to a hospital, linking EMS data to the subsequent ED data enables assessment of the continuum of care and evaluation of health outcomes.

We evaluated the performance of North Carolina’s historical EMS–ED data linkage method, identified opportunities to enhance linkage performance, and determined the proportion of EMS suspected opioid overdose cases that received diagnosis codes for opioid overdose in the ED. We developed an iterative linkage method to maximize the proportion of EMS–ED records that link while limiting false linkages.

## Methods

### Data Sources

The EMS Performance Improvement Center,^6^ the North Carolina EMS data contractor at the time of this study, provided North Carolina EMS data; all licensed EMS agencies in North Carolina used the National EMS Information System (NEMSIS) version 2 in 2017. The North Carolina Disease Event Tracking and Epidemiologic Collection Tool (NC DETECT), North Carolina’s syndromic surveillance system, provided ED data for all 126 civilian acute care hospital–affiliated EDs across North Carolina, excluding military, Veterans Affairs, and tribal hospitals.

Our methods built on an existing deterministic linkage approach implemented in 2009.^7^ Using this historical linkage approach, we linked EMS records to ED records only through an exact match on 3 data elements (date of birth, sex, and destination name [eg, Carolinas Medical Center, Moses Cone Health System, UNC Hospital]) and no more than a 60-minute difference between recorded EMS destination arrival time and first documented ED time. We stored unique identifiers for the linked records from each data set in a separate data file.

### Data Set Creation

We identified all EMS encounters from January 1 through November 30, 2017, by the EMS dispatched-to-the-scene time (N = 1 446 197). We created 3 data sets: (1) all EMS encounters with documented naloxone administration, (2) a 12-county subset of all EMS encounters with naloxone administration, and (3) a simple random sample of all statewide EMS encounters, irrespective of naloxone administration. We used the 12-county subset to evaluate and enhance linkage methods and then applied these methods to the other 2 data sets.

In the first data set, we defined EMS encounters for suspected opioid overdose as encounters with naloxone administration, recorded as either naloxone or Narcan in the free-text medications field. We did not observe misspellings or acronyms among the data elements that noted medications administered. Among the suspected opioid overdoses, we evaluated the linkage performance among encounters recorded as treated and transported by EMS (N = 12 088).

**Figure 1:**
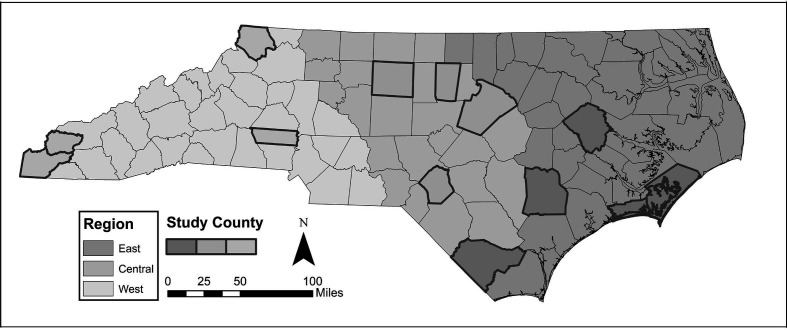
Counties included in the 12-county subset of naloxone administration by emergency medical services, North Carolina, January 1–November 30, 2017. Counties included in the subset by region were Carteret, Columbus, Duplin, and Pitt (eastern region); Guilford, Hoke, Orange, and Wake (central region); and Ashe, Cherokee, Graham, and Lincoln (western region). County-level performance of historical linkage methods ranged from 9.0% to 56.7% in the eastern region, 0% to 80.0% in the central region, and 6.5% to 48.0% in the western region.

**Figure 2:**
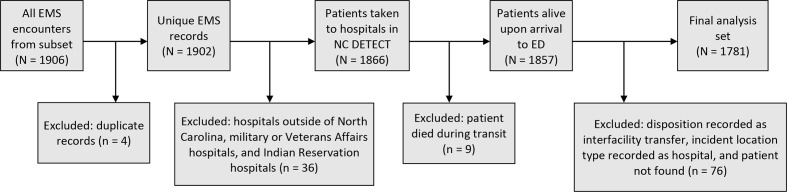
Eligibility criteria applied to a subset of emergency medical services (EMS) encounters before assessing linkage performance, North Carolina, January 1–November 30, 2017. During January 1–November 30, 2017, 1906 EMS encounters with naloxone administration and transport to a hospital occurred in the 12 counties included in the subset. After applying exclusion criteria to identify only EMS encounters that could realistically be linked to the North Carolina Disease Event Tracking and Epidemiologic Collection Tool (NC DETECT) emergency department (ED) records, 1781 EMS encounters were included in the final analytic data set. The same exclusion criteria were then applied to the data set featuring statewide EMS naloxone administration and a simple random sample of all statewide EMS encounters.

To investigate EMS–ED data linkage quality in greater detail, we created a second data set consisting of a 12-county subset of EMS encounters with naloxone administration (N = 1906). We selected 4 counties from each region (eastern, central, and western) representing the range of historical linkage performance (Figure 1). We excluded 125 (6.6%) EMS encounters—76 interfacility transfers, 36 taken to non–NC DETECT hospitals (eg, out-of-state hospitals, military facilities), 9 deaths that occurred during transport, and 4 duplicate records—leaving 1781 EMS encounters with naloxone administration for linkage enhancement (Figure 2).

The third data set included all EMS encounters, not just those with naloxone administration, to assess the performance of the linkage methods overall. From the full set of EMS encounters, we examined a simple random sample of 2500 records.

### Historical Linkage Quality Assessment

We identified EMS encounters that were eligible for linkage, initially defined as EMS encounters with a recorded disposition of “treated and transported” and destination of “hospital.” We later refined eligibility to include only EMS patients taken to North Carolina EDs included in NC DETECT, excluding patients who died during transport or were classified as an interfacility transfer.

Among patients deemed eligible for linkage in the 12-county subset, we identified all unlinked EMS encounters after applying historical linkage methods and developed a gold standard linked data set to characterize reasons for missed linkage. Using available EMS information (age, sex, destination name, destination arrival time, and chief complaint), we manually identified the NC DETECT ED visit that should have linked and recorded the unique ED patient identifier. By comparing linked EMS and ED records in the gold standard data set that were unlinked via historical methods, we characterized the reasons for missed linkage.

### Linkage Enhancement

To improve linkage performance, we reran the historical linkage methods using updated data. The historical linkage performance reflects running the historical linkage methods only once with the first received EMS and ED records; however, ED and EMS data are updated over time, improving the completeness and accuracy of data elements used in the linkage process. We then expanded the hospital name standardization mapping file to address variations among hospital name spellings, misspellings, and abbreviations in the EMS free-text destination name field, overcoming missed linkages resulting from requiring an exact match in the historical linkage methods. We extended the allowed difference between EMS destination arrival time and first documented ED time to 360 minutes (after exploring 90, 120, and 240 minutes). Finally, we implemented an iterative linkage approach. We first ran the linkage using deterministic criteria on date of birth, sex, destination name, and ±360 minutes between EMS and ED times. Then, among the unlinked EMS records, we reran the linkage with deterministic criteria on sex, destination name, and ±60 minutes, while allowing date of birth to vary by 10 days or exactly 1 year (Table 1).

**Table 1:** EMS–ED data linkage optimization methods and iterative improvement, North Carolina, January 1–November 30, 2017

Linkage trial^a^	Data version	Linkage method	Matching criteria	Additions to data	12-county subset (n = 1781)	
					No. (%) linked	No. (%) false links
1	Historical linkage	Deterministic	Date of birth Sex Destination ±60 min	None	1165 (65.4)	28 (1.6)
2	Data with updates as of August 2018^b^	Deterministic	Date of birth Sex Destination ±60 min	None	1349 (75.7)	1 (0.1)
3	Data with updates as of August 2018	Deterministic	Date of birth Sex Destination ±60 min	Added variations to destination hospital names^b^	1417 (79.5)	1 (0.1)
4	Data with updates as of August 2018	Deterministic	Date of birth Sex Destination ±360 min^b^	Added variations to destination hospital names	1593 (89.4)	1 (0.1)
5	Data with updates as of August 2018	Multistage deterministic	Date of birth Sex Destination ±360 min **THEN** Date of birth (±10 d, or −366, −365, 365, or 366 d) Sex/gender Destination ±60 min	Added variations to destination hospital names	1620 (91.0)	1 (0.1)

Abbreviations: ED, emergency department; EMS, emergency medical services.

^a^Efforts to improve the performance of EMS–ED data linkage methods were completed stepwise, using assessments of reasons for missed linkage to inform and prioritize subsequent changes to the methods. These enhancements to linkage methods, and corresponding improvements to performance, are shown here with respect to a 12-county subset of EMS encounters in North Carolina with recorded naloxone administration, selected in a nonrandom fashion from the statewide data to reflect 3 regions in North Carolina (eastern, central, and western), and a range of county-level historical linkage performance within each region.

^b^Changes to the linkage methods to enhance performance were, in order, use of updated date (trial 2), expansion of the hospital name mapping file (trial 3), extending the maximum allowable time difference between EMS and ED times to ±360 minutes (trial 4), and inclusion of an iterative linkage step (trial 5).

### Final Diagnoses in the ED

To investigate whether EMS patients with naloxone administration were diagnosed as opioid overdose or other nonspecific overdoses in the ED, we evaluated the diagnosis codes recorded for linked records. We used the NC DETECT case definition^8^ of opioid overdose (opioid overdose version 1) to evaluate the proportion of linked EMS encounters that received an opioid overdose diagnosis: *International Classification of Diseases, Tenth Revision, Clinical Modification* (ICD-10-CM) codes T40.0-T40.4 for initial or subsequent encounters, excluding underdosing and adverse events.^9^ We categorized ED visits that received at least 1 of these ICD-10-CM codes in any of 16 diagnosis code fields as a confirmed opioid overdose. We then explored diagnoses received by patients who did not receive an opioid overdose diagnosis. We classified patients as having a myocardial infarction diagnosis if they received any ICD-10-CM code I21 for myocardial infarction or I46 code for cardiac arrest, and we identified additional syndromes using ICD-10-CM diagnosis codes and NC DETECT’s prespecified syndrome definitions.^8^ We classified patients into the 0, 1, or >1 syndrome category.

### Statistical Analysis

To evaluate characteristics of the 3 data sets, we calculated the proportion of EMS encounters recorded as male and the mean age and standard deviation (SD). With each update to the linkage methods, we calculated the proportion of EMS encounters linked and, for the 12-county gold standard data set, the proportion of false linkages. In addition, we compared the average age and sex distribution among linked and unlinked data sets to assess potential bias in linkage methods. We calculated the positive predictive value (PPV) of using EMS naloxone administration to identify overdose cases as the number of patients with the relevant codes or syndromes divided by the total number of EMS encounters with naloxone administration. We performed data linkage using Microsoft SQL Server 2014, we conducted statistical analyses using SAS version 9.4 (SAS Institute Inc), and we created maps using ArcGIS version 10.1 (Esri). Because this project was a quality improvement project for public health surveillance, we did not seek or require institutional review board approval.

## Results

### Characteristics

In the 12-county subset, the average patient age was 45.0 years (SD = 18.8), and 58.4% (1040 of 1781) of patients were male (Table 2). In the full data set, the average age was 45.0 years (SD = 18.5), and 57.7% (6582 of 11 412) of patients were male. In the simple random sample of all eligible EMS encounters, the average age of patients was 56.8 years (SD = 23.6), and 50.7% (1169 of 2305) of patients were male.

**Table 2:** Characteristics and enhancement of EMS–ED data linkage performance among 3 data sets of EMS encounters, North Carolina, January 1–November 30, 2017

Characteristic	12-county subset^a^ of EMS encounters with naloxone administration (n = 1906)	EMS encounters with naloxone administration, statewide (N = 12 088)	Statewide sample of all EMS encounters (SRS; N = 2500)^b^
Linkage-eligible EMS encounter, no. (%)^c^	1781 (93.4)	11 412 (94.4)	2305 (92.2)
Age, mean (SD), y^d^	45.0 (18.8)	45.0 (18.5)	56.8 (23.6)
Male, no. (%)^d^	1040 (58.4)	6582 (57.7)	1169 (50.7)
Initial linkage performance, no. (%)	1165 (65.4)	5378 (47.1)	1456 (63.2)
Final linkage performance, no. (%)	1620 (91.0)	10 399 (91.1)	1934 (83.9)
Linkage improvement, percentage-point difference	25.6	44.0	20.7

Abbreviations: ED, emergency department; EMS, emergency medical services; NC DETECT, North Carolina Disease Event Tracking and Epidemiologic Collection Tool; SRS, simple random sample.

^a^Subset includes all EMS encounters with naloxone administration and transport to hospitals in NC DETECT from 4 North Carolina counties from each region: eastern (Carteret, Columbus, Duplin, and Pitt), central (Guilford, Hoke, Orange, and Wake), and western (Ashe, Cherokee, Graham, and Lincoln).

^b^A simple random sample of 2500 EMS encounters was drawn from all EMS encounters that occurred in North Carolina during the study period. This set was then restricted to only those deemed eligible for linkage.

^c^Linkage eligibility was restricted to EMS encounters in NC DETECT in which the patient was treated and transported to hospitals, was alive upon arrival to the ED, and was not an interfacility transfer.

^d^Demographic characteristics of only the linkage-eligible population.

### 12-County Subset: Historical Linkage Performance, Eligibility, and Key Factors

The historical linkage performance among the 12-county subset was 65.4% (1165 of 1781). During evaluation of the 616 EMS encounters not linked using the historical methods, we were unable to locate an ED record for 35 EMS encounters and therefore unable to characterize the reason for the missed linkage. In addition, we could not identify a reason for missed initial linkage for 184 encounters, all of which successfully linked when running the historical methods on updated data.

Among the remaining 397 unlinked EMS records, 320 (80.6%) had a single variable responsible for missed linkage and 49 (12.3%) had 2 variables responsible for missed linkage. The most common reason was a >60-minute difference between the EMS handoff time and ED initial documented time (n = 164 encounters). Upon further inspection, we found that some hospitals were erroneously sending updated ED initial documented times. For some, but not all, encounters with discrepancies in EMS–ED time, we easily identified this problem when comparing ED initial documented time with time stamps in triage notes. One example was a 58-year-old patient whose record indicated an ED initial documented time of 5:40 am, but triage notes revealed the first evaluation of the patient occurred at 1:11 am. The EMS destination arrival time for this patient was 1:22 am, within the 60-minute window allowed by historical linkage methods. We additionally identified 133 EMS–ED records with errors in the recorded date of birth in 1 or both data systems. Many of the discrepancies between the listed date of birth in the EMS and ED data occurred because of typos or miscommunications, such as an exact 1-year difference. The free-text capture of destination names in the NEMSIS version 2 data structure produced numerous variations in how the same hospital was recorded for EMS encounters. For example, EMS providers used 16 variations to record Frye Regional Medical Center (eg, Frye, Frye Medical Center, Frye Reg Med Ctr). We then used a hospital name–mapping process to standardize hospital names. Despite including 2182 name variations for the 126 hospitals in NC DETECT, 113 EMS encounters still did not link to the corresponding ED visit record because of problems with the recorded destination name. Discordantly recorded sex affected only 7 EMS–ED cases.

### Linkage Methodology Enhancement

**Figure 3:**
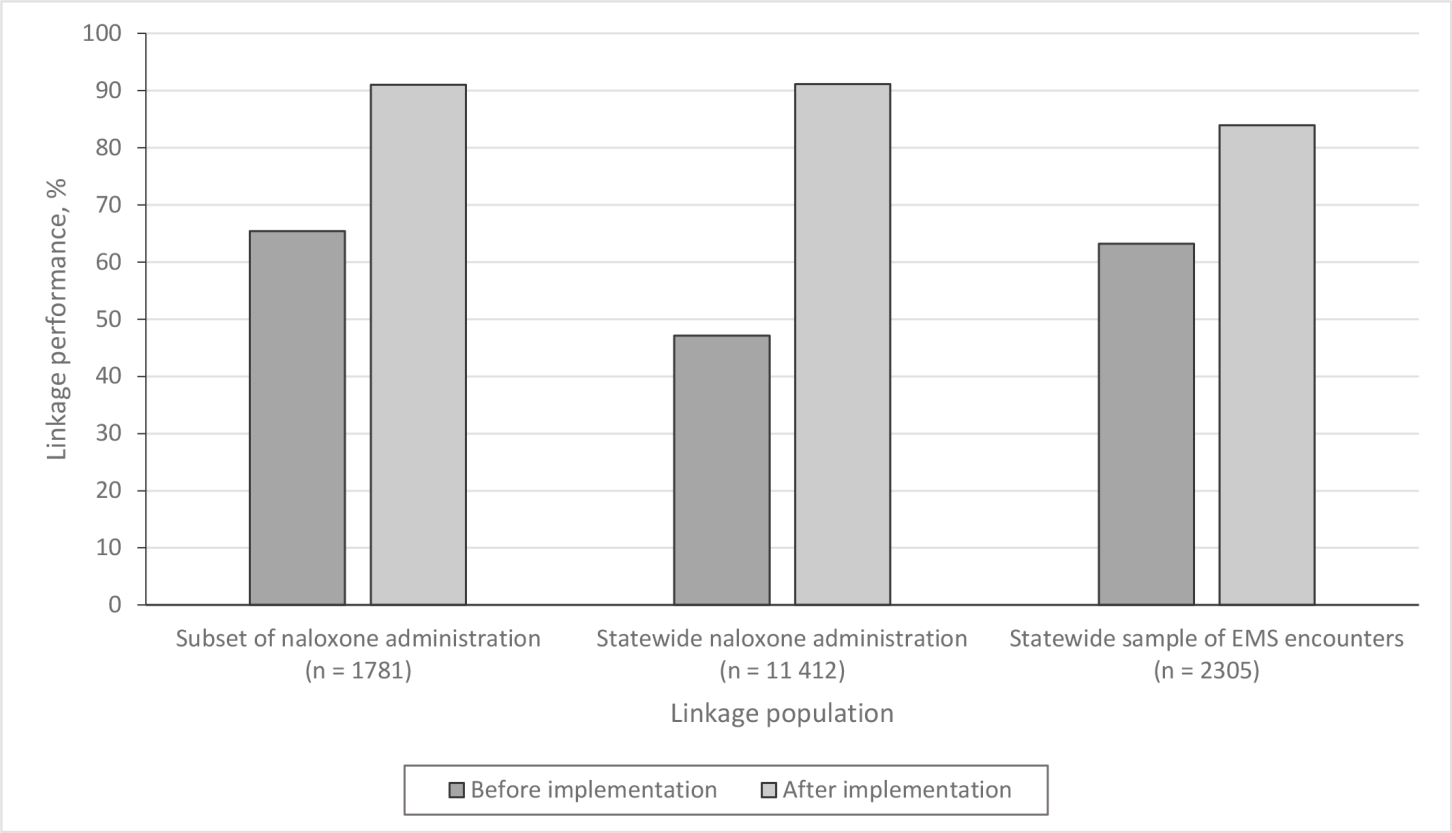
Improvement of EMS–ED linkage performance through implementing modifications to historical linkage methods and adding an iterative linkage step, North Carolina, January 1–November 30, 2017. Linkage eligibility was defined as EMS encounters transported to North Carolina EDs with data included in NC DETECT. The subset of naloxone administration has EMS data from 12 North Carolina counties, selected nonrandomly from the statewide data to reflect 3 regions (eastern, central, and western), and a range of county-level historical linkage performance within each region. Abbreviations: ED, emergency department; EMS, emergency medical services; NC DETECT, North Carolina Disease Event Tracking and Epidemiologic Collection Tool.

By using updated EMS and ED data, linkage performance improved by 10.3 percentage points to 75.7% (1349 of 1781). After updating the destination names, extending the time to ±360 minutes, and adding a multistage method allowing for flexibility on date of birth matching, the enhanced linkage performance improved by 25.6 percentage points to 91.0%. We observed no differences in average age or sex distribution between the linked and unlinked records. In addition, neither extending EMS–ED time differences nor applying less strict matching criteria on date of birth resulted in more false linkages (Table 1; Figure 3).

The enhanced linkage methods improved performance from 47.1% (5378 of 11 412) to 91.1% (10 399 of 11 412) for statewide EMS encounters with naloxone administration and from 63.2% (1456 of 2305) to 83.9% (1934 of 2305) in the statewide sample of all linkage-eligible EMS encounters (Table 2; Figure 3).

### Overdose Diagnoses

Among the 10 399 EMS encounters with naloxone administration that linked to ED records, 27.2% (n = 2833) included an ED discharge diagnosis code for opioid overdose. Among linked EMS–ED records with an opioid overdose diagnosis code, the average patient age was 35.2 years (SD = 12.3); the average age among linked records without an opioid overdose diagnosis code was 48.2 years (SD = 19.0). Exploratory analyses of the 7566 linked records without a diagnosis code for opioid overdose revealed that 3384 (PPV = 44.7%) had a medication or drug overdose based on an ICD-10-CM code or overdose keyword, and 2121 (PPV = 28.0%) had a medication or drug overdose based on an ICD-10-CM code only. The most common non-opioid overdose diagnosis group observed was myocardial infarction (13.7%; 1038 of 7566). Combining linked records classified as opioid overdoses by ED diagnosis and those with medication or drug overdose based on ICD-10-CM codes or keywords, we identified 6217 opioid overdose patients (PPV = 59.8%).

## Discussion

### Importance of Creating a Gold Standard Data Set

Developing a gold standard data set was critical to improving our linkage performance. By evaluating EMS–ED records that should link, we identified the most common characteristics leading to linkage failure, which informed modifications to the linkage methods and our iterative strategy. Using this knowledge, we took steps to improve data quality for future linkage. Key improvement steps include postponing linkage to use the most complete data available, prohibiting updates to the ED initial documented time to minimize time differences between EMS and ED records, and standardizing hospital destination names.

### Approaches to Data Linkage

We used deterministic linkage methods to link de-identified records. Other efforts to link EMS data to ED data have used probabilistic and machine learning methods, with varying degrees of success. Probabilistic linkage of EMS and inpatient records among transported cardiac arrest patients in Michigan produced an estimated linkage sensitivity of 48.2%.^10^ EMS data linked with the state trauma registry in Oregon achieved 99.6% sensitivity using probabilistic methods.^11^ Similar to our linkage work in North Carolina, linkage activities in Michigan and Oregon had limited identifiers available (eg, sex, age, date of birth). Data linkage using patient identifiers, such as names or social security numbers, improves performance compared with data sets that lack patient identifiers.^12,13^

By implementing an iterative deterministic linkage approach, we substantially improved the performance of EMS–ED linkage in North Carolina. The iterative approach achieved additional linkages while limiting the number of records passing through less stringent linkage requirements, thereby reducing the risk of false linkages. This work improved upon a historical linkage approach and can be applied to other data for which probabilistic linkage methods are not desirable.

### ED Outcomes for EMS Naloxone Patients

We leveraged improved linkage performance to evaluate final ED diagnoses among patients transported by EMS with suspected opioid overdose, revealing that only 27.2% of EMS cases ultimately received an ICD-10-CM code for opioid overdose. Research on using naloxone administration by EMS as a proxy for opioid overdose found moderate PPV (60.0%) for accurately identifying “known or presumed opioid patients” after medical record review.^5^ Among EMS records with naloxone administration linked to ED records from 3 hospital EDs in the same health care system, 48% were determined to be opioid overdose by medical record review.^14^ Our study found low PPV (27.2%) for identifying opioid overdose cases by diagnosis codes alone but a similar PPV (59.8%) when using EMS naloxone administration to identify ED visits diagnosed with any medication or drug overdose by diagnosis codes or keywords. Considering the challenges of using naloxone administration to identify true opioid overdose cases, we must develop a better understanding of who is receiving naloxone from EMS and what common conditions are being misclassified as suspected opioid overdose.

### Impact of EMS–ED Data Linkage

Transferring key prehospital information to ED health care providers improves care for high-risk patients through the determination of appropriate treatment plans.^15,16^ By extension, linking EMS and ED data for surveillance and research purposes allows a retrospective assessment of associations between patient outcomes in the ED and interventions used during the EMS response. Findings can inform improvements to prehospital care.

### Limitations

This study had several limitations. First, available EMS data elements limited our ability to identify linkage-eligible EMS encounters. We relied on a destination type of “hospital” to determine baseline linkage eligibility, but we could not determine whether the patient was actually transported to and seen in the ED. For example, an EMS patient transported to the ED may leave the ED before being seen by a health care provider, resulting in limited ED visit information. Alternatively, the EMS patient may be directly admitted to a different hospital department, resulting in no ED record to link. Second, the counties included in the gold standard data set were not randomly selected, and this selection method may have introduced bias. However, the major update to linkage methods based on the gold standard data set was widening the allowable difference in time in EMS and ED times, and the 12-county subset included the county with the greatest number of records with large EMS–ED time differences; as such, this sample provided the best information for enhancing linkage methods. Third, a single reviewer created the gold standard data set. A lack of multiple reviewers introduces the potential for missing true linkages, incorrectly designating why a link was missed, or incorrectly identifying the correct ED link to an EMS record. We do not believe our medical record review strategy created substantial error, because the proportion of EMS records not matched to an ED record was small (2.0%), detailed information (narratives among EMS, triage notes among EDs) was used to determine appropriate pairs, concordance between linkage variables was not subjective, and categorization of reasons for missed linkage was reviewed a second time for correctness after all EMS–ED pairs were identified in the gold standard data set. Finally, to determine whether a naloxone-administered patient was a true opioid overdose case, we relied on secondary diagnosis code data, rather than full medical record review, which was impossible for our population-based study. The use of only diagnosis code data may have resulted in an underestimate of the true number of opioid overdose cases.

## Implications

Linkage methods must continue to be assessed, as updates and new versions of NEMSIS and ED data may affect data quality and produce additional data elements that could enhance linkage performance. For example, NEMSIS version 2.0 destination type “hospital” is expanded in NEMSIS version 3.4 to include “treated and transported to hospital ED” or “treated and transported to hospital non-ED bed,” among other options. This increased granularity may improve our ability to identify linkage-eligible records. The average amount of time from the original submission of EMS and ED records to when records are no longer updated should be investigated to inform when linkage should be completed and provide optimal balance between linkage timeliness and performance. We continue to explore additional iterative steps to maximize linkage performance.

Linking EMS and ED data allows for a continuum of care assessment and enhances research on procedures and health outcomes. This information is critical for public health surveillance of opioid overdose and will improve quality of care. Efforts in North Carolina demonstrate that, with a modest investment of resources, improvements in data linkage are possible and allow a greater understanding of EMS cases suspected of opioid overdose at the scene and then transported to and treated in EDs. Timely and accurate data in these systems will increase our understanding of the scale of the current opioid overdose crisis.
